# Enzymatic Removal of Diclofenac and Aceclofenac from Water by Soybean Peroxidase

**DOI:** 10.3390/molecules30081817

**Published:** 2025-04-18

**Authors:** Sara Pishyar, Samira Narimannejad, Keith E. Taylor, Nihar Biswas

**Affiliations:** 1 Department of Civil and Environmental Engineering, University of Windsor, Windsor, ON N9B 3P4, Canada; pishyar@uwindsor.ca (S.P.); samiran@uwindsor.ca (S.N.); biswas@uwindsor.ca (N.B.); 2 Department of Chemistry and Biochemistry, University of Windsor, Windsor, ON N9B 3P4, Canada

**Keywords:** wastewater treatment, enzymatic treatment, emerging contaminants, soybean peroxidase

## Abstract

Pharmaceuticals are a class of emerging contaminants that have been widely detected in wastewater treatment facilities’ influent and effluent. They threaten the environment and non-target life. Thus, a promising treatment method, soybean peroxidase (SBP; EC 1.11.1.7), which catalyzes the oxidation of phenolic and anilino donors in the presence of hydrogen peroxide, was investigated as a treatment method. The aim was to remove two non-steroidal anti-inflammatory drugs, diclofenac (DCF) and aceclofenac (ACF), from synthetic wastewater via enzymatic oxidation, oligomerization, and precipitation. SBP can be extracted from soybean hulls, a byproduct of the soybean industry. DCF (0.10 mM) and ACF (0.10 mM) were amenable to SBP-catalyzed removal under the optimal operational parameters of pH 5 and 4; hydrogen peroxide: 0.40 and 0.45 mM; and minimum effective enzyme concentration: 0.15 and 0.60 U/mL, respectively. The initial first-order rate constant and half-life of each substrate were also determined under the established optimum conditions. Under these optimum conditions, the half-lives for DCF and ACF were 1.43 ± 0.01 and 0.84 ± 0.05 min, respectively. The results demonstrated that SBP is a robust enzyme that can achieve more than 95% removal for both compounds. Mass spectrometric analysis of the enzymatic treatment products of DCF revealed the formation of an oxidative tetramer. The SBP-catalyzed reaction is a highly effective method for removing DCF and ACF from synthetic wastewater, highlighting its potential for environmental cleanup of pharmaceutical contaminants.

## 1. Introduction

Emerging contaminants (ECs) are newly recognized potential contaminants that have negative influences on the environment and/or human health, but that are not yet regulated [1]. Pharmaceuticals are a class of emerging contaminants, and the widespread occurrence of these substances in surface water and effluents is currently of concern in water quality regulation due to the potential threats they provide to human health and the environment. Non-steroidal anti-inflammatory drugs (NSAIDs) such as DCF (2-(2,6-dichloranilino) phenylacetic acid; DCF) and ACF ((2-{2,6-dichlorophenyl) amino} phenylacetooxyacetic acid; ACF), are among the most frequently used pharmaceuticals in both human and veterinary medicine. In the US alone, it is estimated that more than seventy million medical prescriptions are written, and more than 30 billion dosages are utilized annually [2]. The European Union is the second-largest user of these medications, with 50–150 g of NSAIDs per person annually, or around 24% of the global total [3]. In recent years, several studies have documented the widespread, ongoing, and rising presence of NSAIDs in marine, surface, and ground waters, as well as sewage sludge and effluent and industrial and hospital effluents [4,5,6,7,8,9]. NSAIDS are both directly and indirectly released into the environment. Wastewater effluents from various sources such as businesses, healthcare facilities, and wastewater treatment plants, often contain such compounds, substances that conventional sewage treatment methods cannot effectively remove [10]. For example, DCF has been reported in sewage treatment plant effluent at 349 and 237 ng/L for the parent compound and its 4′-hydroxy metabolite, respectfully (removal rate by sludge, 21%), and ACF was detected at 31 and 59 ng/L as the parent and 4′-hydroxy metabolite, respectively. The same study reported that ACF was relatively easily hydrolyzed to DCF by the activated sludge process [11]. A more recent study reported DCF in sewage treatment plant effluent as 647 ng/L [12]. These compounds are a significant environmental issue with harmful consequences for the ecology, flora, and fauna, as well as humans. NSAIDs, even at concentrations as low as ng to ug/L, can negatively impact the health, behavior, reproduction, and survival of aquatic species across multiple trophic levels [13,14,15].

**Figure 1:**
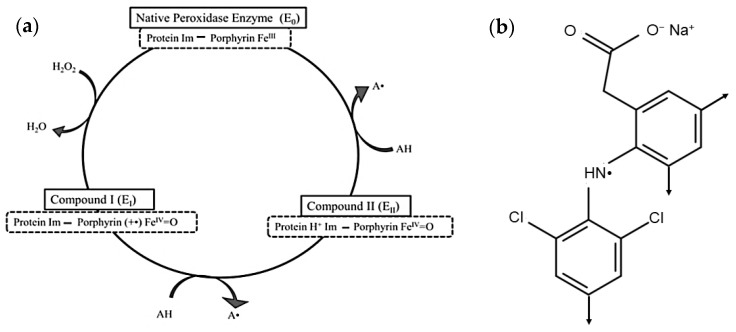
(**a**) The peroxidase cycle for oxidative polymerization of arylamines, where Protein Im—Porphyrin represents the imidazolyl side-chain of a histidine residue of the enzyme protein complexed to the porphyrin iron (III); in Compound I, the preceding complex has been 2-electron oxidized by H_2_O_2_ to a cation radical with iron in the Fe^IV^=O state; in Compound II, the preceding complex, Compound I, has been 1-electron reduced to the cation (protonated imidazolyl ring) with iron in the Fe^IV^=O state and with concomitant 1-electron oxidation of an arylamine, AH, to the free radical, A**^.^**, the DCF radical, (**b**), in this work; Compound II returns to the resting enzyme state by 1-electron reduction with a second concomitant 1-electron oxidation of AH to A. (**b**) DCF radical (A^.^) in (**a**) with arrows indicating *ortho*- and *para*-C–C coupling sites for dimer formation in a subsequent peroxidase cycle.

Enzymatic treatment has been applied successfully as an effective wastewater treatment method for various emerging contaminants in water and wastewater including NSAIDs. Soybean seedcoat peroxidase (SBP; phenolic donor:hydrogen-peroxide oxidoreductase; EC 1.11.1.7) has demonstrated the ability to catalyze the oxidation of a variety of different compounds. The peroxidase cycle is shown in Figure 1a [16]. The reactive radicals generated, A**^.^** in Figure 1a, couple non-enzymatically by C-C coupling (N-C is possible but unlikely due to steric crowding) with *ortho*-, *para*-orientation to form dimers, Figure 1b, that grow analogously with additional peroxidase cycles until they reach their solubility limit and precipitate and can be removed by filtration or sedimentation. In some cases, literature references are given below for laccase-catalyzed reactions of arylamine substrates because that enzyme class generates the same aryl free radicals, which then couple non-enzymatically as for the peroxidase-catalyzed reactions [17].

This study investigated the feasibility of enzyme-catalyzed treatment of two selected non-steroidal anti-inflammatory drugs (NSAIDs), DCF and ACF, in synthetic wastewater using soybean peroxidase (SBP). The experimental approach involved optimizing key operational parameters, pH, hydrogen peroxide concentration, and enzyme activity, to achieve a removal efficiency of ≥95%. The enzymatic reactions were conducted under controlled conditions, and the conversion kinetics were analyzed by determining the initial first-order rate constants and half-lives of each substrate. Furthermore, mass spectrometry was employed to identify plausible oligomerization products formed during the enzymatic treatment. The findings demonstrated that SBP effectively catalyzes the oxidation of DCF and ACF, leading to the formation of oligomeric products that precipitate from the solution. These results highlight the potential of SBP as a sustainable and efficient biocatalyst for the removal of pharmaceutical contaminants from wastewater, offering an environmentally friendly alternative to conventional treatment methods.

## 2. Results and Discussion

This study assessed the possibility of utilizing SBP-catalyzed reactions to convert two NSAIDs, DCF and ACF, and optimized crucial operational factors, pH, H_2_O_2_ concentration, and enzyme activity, with the goal of achieving a conversion efficiency of ≥95%. Reaction times of experiments based on optimal parameters were measured to determine the time to reach 95% conversion and to find the initial first-order reaction rate constants and half-lives of substrates. The reactions were operated under optimized conditions to identify possible products by mass spectrometric analysis. Results in the following sections clearly indicate ACF and DCF are relatively good substrates of SBP, amongst the few secondary amines so discovered with peroxidases and laccases [17,18].

### 2.1. Effect of pH

Optimum pH was determined under stringent conditions with limited SBP, resulting in incomplete substrate removal for clear pH effect distinction. A broad pH range was initially explored, followed by narrower range experiments. Hydrogen peroxide concentration at this stage was taken as 1.5–2.0 times the substrate’s molar concentration of 0.10 mM, based on substrates studied previously in the lab [16]. Experiments were performed in triplicate at room temperature (22 ± 1 °C).

**Figure 2:**
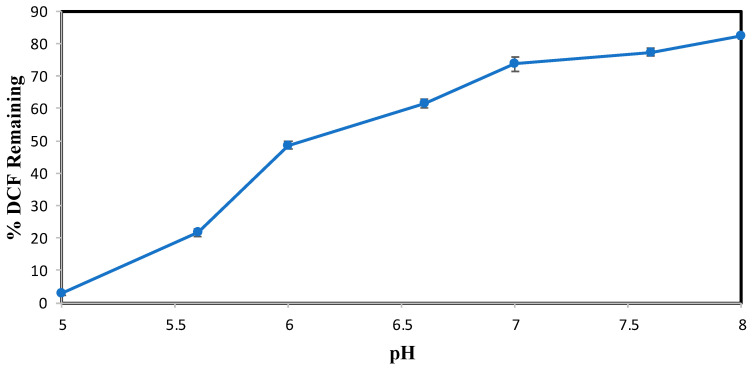
DCF pH Optimization. (0.10 mM DCF, 0.40 mM H_2_O_2_, 0.15 U/mL SBP, 3 h reaction, room temperature). Reactions conducted in triplicate; error bars that are not visible are smaller than the symbols.

The effect of pH on the conversion of DCF after 3 h of enzymatic treatment is shown in Figure 2. Subsequent experiments were analyzed by HPLC over a narrower pH range of 3.0–8.0 and at an SBP activity of 0.15 U/mL, with 0.40 mM hydrogen peroxide. Figure 2 indicates that the most effective pH for DCF enzymatic conversion is 5.0, which showed 4% remaining with 0.15 U/mL SBP. Above this pH value, the enzymatic activity decreased drastically, causing a reduced efficiency in catalytic conversion. However, DCF precipitated in reaction mixtures below pH 5.0 and in the no-SBP control. This could be due to the pK_a_ of DCF, 4.18, [19], the protonated carboxyl form being much less soluble than the carboxylate. Therefore, at pH values < 5.0, enzymatic reactions of DCF could not be conducted because DCF predominantly exists in its neutral form, exhibiting lower solubility and precipitating under these conditions [20,21]. For example, at pH 5.0 100% of 0.10 mM DCF was soluble, whereas at pH 3.0, only 22% of the compound remained in the buffered reaction solution. Hence, pH 5.0 was chosen as the most effective for DCF. This choice is further supported by the pH behavior of ACF, shown below, for which precipitation did not occur at pH values < 5.0.

**Figure 3:**
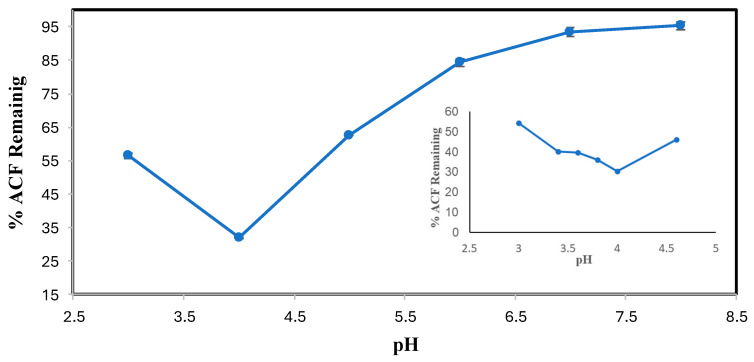
ACF pH optimization. (0.1 mM ACF 0.4 mM H_2_O_2_, 0.15 U/mL SBP, 3-hour reaction, room temperature). Reactions conducted in triplicate; error bars that are not visible are smaller than the symbols.

The results of the ACF pH optimization are shown in Figure 3. Solubility was not an issue, thus it could be studied to lower pHs, from which it is seen that ACF (pK_a_ = 4–5) was best transformed in the pH range of 3.0–4.6 with the optimum pH being 4.0 (Figure 3 inset). For both DCF and ACF, conversion was increasingly poorer as the pH increased beyond the pK_a_, likely because of an increase in ionization of the carboxyl group to the anionic form of the substrate that is not able to participate in the peroxidase cycle [22]. The bell-shaped pH dependence for ACF shows a variation in the ionization state of the enzyme’s catalytic residues and/or the ionization state of the substrate [23]. 

The pH optima for the DCF and ACF show that SBP converts these anilines under mildly acidic conditions, which has been observed for other anilino compounds such as phenylenediamines, benzidine, and 4-chloro-*o*-toluidine, which showed similar trends, exhibiting respective optima at pH 4.5–5.6, 5.0, and 4.4–5.0; at pH > 7.0, there was almost no removal of these compounds [24,25,26]. The same pH sensitivity was found for the laccase-catalyzed conversion of DCF [27,28].

### 2.2. Effect of SBP Activity

**Figure 4:**
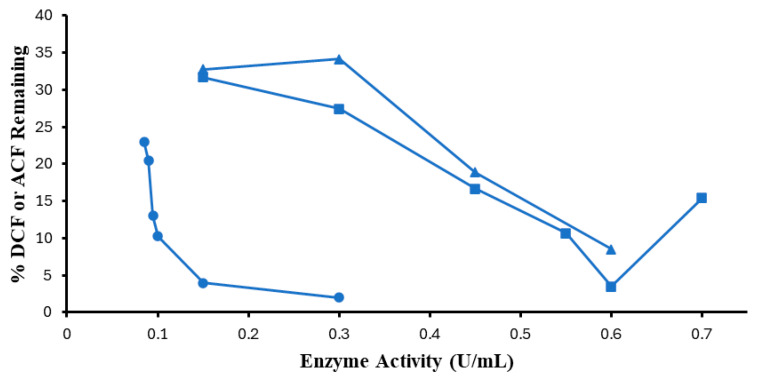
Enzyme activity optimization. (0.1 mM DCF, pH 5.0, 0.40 mM H_2_O_2_, ●; 0.1 mM ACF, pH 4.0, 0.40 mM H_2_O_2_, ▲; 0.10 mM ACF, pH 4.0, 0.45 mM H_2_O_2_ ■; 3 h reaction, room temperature). Reactions conducted in triplicate; error bars that are not visible are smaller than the symbols.

The minimum SBP activities needed to achieve 95% conversion of substrates were sought for 0.10 mM DCF and ACF at the most effective pH values determined above and non-limiting H_2_O_2_ concentrations, as shown in Figure 4. For ACF, 95% removal was not reached with 0.40 mM H_2_O_2_, therefore, the SBP sensitivity experiment was repeated at 0.45 mM H_2_O_2_. The minimum effective SBP activity for 0.10 mM DCF was found to be 0.15 U/mL, at which there was 96% conversion, which is consistent with the 0.10 and 0.15 U/mL required for optimal SBP conversion of 0.1 mM of 4,4′-methylenebis(2-chlororaniline) and 4,4′-thiodianiline, respectively [29]. Further increase in enzyme activity had a very slow response in terms of the percentage of removal, hence, using 0.30 U/mL of SBP resulted in ~98% removal of substrate from the solution. Thus, a doubling of the enzyme activity only contributed an additional ~2% increase in efficiency. Very high removal efficiency (>96%) would come at an increase in enzyme cost.

In stark contrast, for 0.10 mM ACF, as shown in Figure 4, the most effective SBP concentration was 0.60 U/mL which achieved 91.5% removal (8.5% remaining); i.e., four times the amount of enzyme was needed for removal of the same amount of DCF. Higher SBP activity leads to poorer conversion of this sluggish substrate, likely due to competing pathways for enzyme inactivation in the presence of excess peroxide [30]. As seen in Figure 4, this could be remedied by a small increase in the peroxide concentration, in accordance with the following section. Since the two substrates are identical in the diphenylamine fragment, the part that undergoes 1-electron oxidation on nitrogen, it may be speculated that the four-fold difference between them in enzyme requirement may be due to how the enzyme accommodates the carboxymethyl ‘side-chain’, with the extended side-chain of ACF fitting less well onto the enzyme surface.

### 2.3. Hydrogen Peroxide Sensitivity

**Figure 5:**
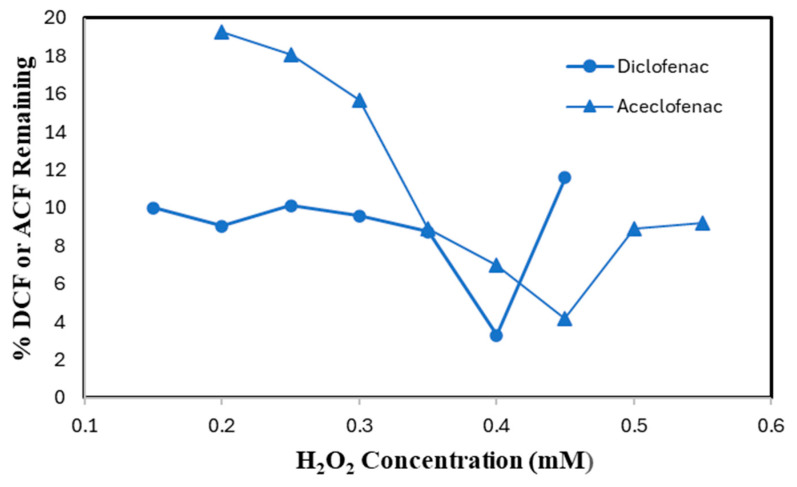
H_2_O_2_ Optimization. (0.10 mM DCF, pH 5.0, 0.15 U/mL SBP; 0.10 mM ACF, pH 4.0, 0.60 U/mL SBP; 3 h reaction, room temperature). Reactions conducted in triplicate; error bars that are not visible are smaller than the symbols.

SBP-catalyzed conversion of DCF and ACF was conducted to optimize H_2_O_2_ concentration at the pre-determined most effective pH values and minimum effective SBP activities. For 0.10 mM DCF and ACF, the best performance was observed at 0.40 mM of H_2_O_2_ with a minimum of 3.4% of substrate remaining and 0.45 mM H_2_O_2_ concentration (4.2% remaining), respectively, as shown in Figure 5. Increasing the concentration of H_2_O_2_ over 0.40 mM for DCF and 0.45 mM for ACF, resulted in a lower removal efficiency. This might be due to inactivation of SBP through the formation of compound III (reversibly catalytically inactive) in the presence of excess H_2_O_2_, or conversion to the irreversibly inactive derivative P-670 [16].

Enzyme optimization was repeated with optimum hydrogen peroxide for ACF. Comparing the optimum point with 0.45 mM H_2_O_2_ with 0.40 mM, a small improvement was observed (3%) in the removal efficiency, as shown in Figure 4 above.

### 2.4. Reaction Times

**Figure 6:**
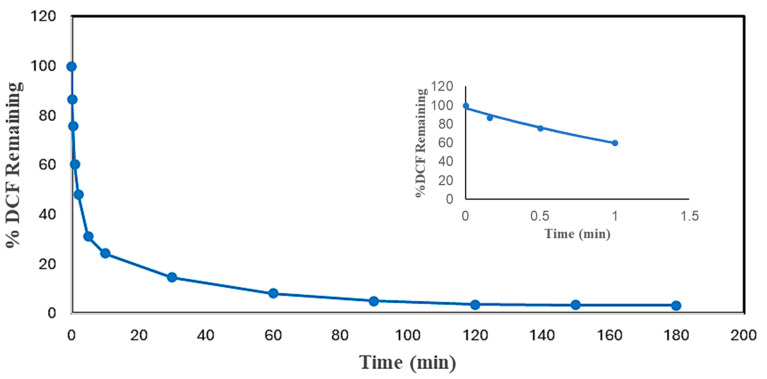
Time dependence of conversion of DCF in optimal conditions batch reactor. (0.10 mM DCF, pH 5.0, 0.40 mM H_2_O_2_, 0.15 U/mL SBP, 3 h reaction, room temperature). Inset shows first-order fit of the first 4 data points: % DCF Remaining = 96.946e^−0.484*t*^, R^2^ = 0.9777.

**Figure 7:**
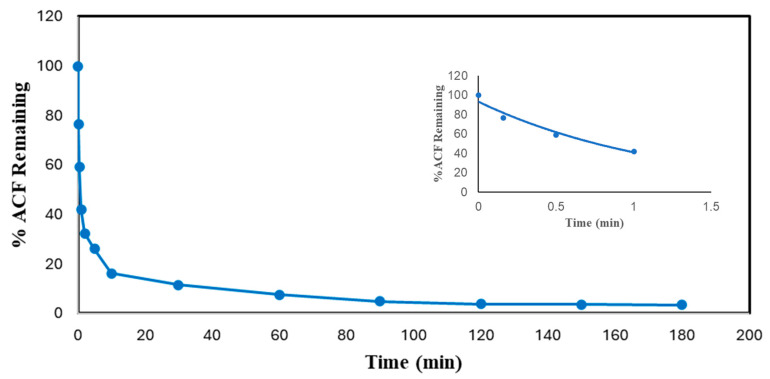
Time dependence of conversion of ACF in optimal conditions batch reactor. (0.1 mM ACF, pH 4.0, 0.45 mM H_2_O_2_, 0.60 U/mL SBP, 3 h reaction, room temperature). Inset shows first-order fit of the first 4 data points: % ACF Remaining = 93.02e^−0.823*t*^, R^2^ = 0.9591.

Reaction time is one of the essential parameters for treatment plant design. It has a close association with the reactor volume and overall cost of the plant. Thus, measurements of the reaction times of experiments were carried out to find the minimum reaction time and reduce the cost of enzymatic treatment while achieving more than 95% pollutant conversion. The reaction times of DCF and ACF were determined under the most effective conditions, as shown in Figure 6 and Figure 7. The initial pseudo-first-order rate constants, *k*, and half-lives of DCF and ACF were calculated based on Equations (1) and (2). It was expected that these reactions would not remain pseudo-first order for very long because of the loss of enzyme activity and/or the progressive consumption of the non-monitored substrate, hydrogen peroxide. Nevertheless, in the absence of a detailed kinetic study, this rate constant will be useful in comparing the reactivity of the enzyme with various substrates.

(1) $$C=C_{0}e^{-kt}$$

(2) $$t_{\frac{1}{2}}=\frac{\ln\left(2\right)}{k}$$

As can be seen from Figure 6 and Figure 7, both compounds reached more than 95% conversion; almost 86% of DCF was converted within the first 30 min, but the conversion of the rest (11%) took 2.75 h; ACF reached 88% conversion at 30 min, and an only 8% improvement in treatment efficiency ensued after the first 30 min, through to the end. The slowing reaction rate is logical since the active enzyme activity decreased with time, attributed to progressive enzyme inactivation [24]. Similar results were found in the SBP-catalyzed removal of 4-chlorophenol, 3-hydroxyquinoline, arylamines, azo dyes, and pesticides [29,31,32,33].

Based on Equations (1) and (2) and the fitted-line equations as shown in the insets of Figure 6 and Figure 7, the initial rate constants for DCF and ACF were 0.484 ± 0.010 and 0.823 ± 0.020 min^−1^, respectively; thus, the corresponding half-lives were 1.43 ± 0.01 and 0.84 ± 0.05 min, respectively. When normalized with respect to enzyme activity, the half-lives were 0.22 ± 0.02 min per U/mL of SBP for DCF and 0.49 ± 0.01 min per U/mL of SBP for ACF. Thus, the normalized initial catalytic reaction rate constant for DCF is approximately twice as fast as that of ACF. This kinetic advantage of DCF parallels that of substrate turnover reflected by the enzyme requirement for 95% conversion noted earlier.

Table 1 summarizes the normalized half-lives of various SBP substrates studied in this laboratory and substrates of this study with respect to the optimum enzyme found in the literature [34]. From the table, it may be seen that DCF and ACF are among the fastest-acting substrates (normalized t_½_ <0.5 min.U/mL) in the comparison.

### 2.5. Preliminary Product Identification Using Mass Spectrometry

Free radicals, generated through enzymatic oxidation, diffuse away from the enzyme and undergo non-enzymatic coupling in solution. As their solubility limit is reached, the oligomers precipitate, ending the polymerization process. Resonance-stabilized radical structures result in multiple coupling sites (C-C, C-O, N-N, N-C) with *ortho*- or *para*-orientation. These loci of unpaired electron density in the radicals produce diverse polymerization products, including oxidative dimer, trimer, etc. (M_2_-2, M_3_-4, etc.). In this study, MS analysis was conducted for DCF using the electro-spray ionization technique (mainly in positive-ion mode, thus, protonated forms of the products were frequently detected; the negative-ion mode was also available and occasionally used). Following optimal enzymatic treatment of the substrates, the filtered reaction mixture and standards were analyzed using high-resolution instruments at Queen’s University. MS masses are mass-to-charge ratios (*m*/*z*; z values are invariably 1 as operated). The isotope abundance was considered during the analysis to support the assigned formulae. The following symbols have been used for the structures proposed: M, standard; MH, protonated standard (protonation occurs in the instrument in positive-ion mode); ^13^C-MH, protonated natural abundance ^13^C-isomer of the standard; M_2_H-2, protonated oxidative dimer; M_3_H-4, protonated oxidative trimer; and M_4_H-6, protonated oxidative tetramer. Free acid and dechlorination formation are denoted here by -Na and -Cl, respectively. Thus, M_4_H-6-Na-Cl denotes an oxidative tetramer as the dechlorinated free acid. Table 2 provides a summary of the MS results, including peaks that were confirmed (noted with an asterisk *) and their assignments, as well as the masses not found, but that were sought as plausibly expected.

Table 2 demonstrates that compounds were only found in their protonated forms, including the residual starting material, DCF. The expected oxidative tetramer was found along with the protonated free acids (MH-Na+H, M_4_H-6-4Na+H) that confirm the loss of Na under reaction conditions to provide a free carboxylic acid functional group (-COOH). With DCF, the nitrogen-centered radical is unlikely to undergo coupling due to steric hindrance at that position; rather, delocalization of radical electron density onto the *ortho*- and/or *para*-positions and thence C-C coupling would be expected in the tetramer. This regiochemistry cannot be determined by MS.

The protonated oxidative tetramer, which is partially dechlorinated, (M_4_H-6-3Cl) was also found. The dechlorination process was not due to the MS conditions; it must have occurred during the enzymatic reaction process since the standard does not show a loss of Cl in the MS analysis. Furthermore, the no-SBP and no-H_2_O_2_ controls showed no DCF conversion by HPLC. Loss of chlorine was reported previously for chlorinated phenols and their binding to humic acid during enzymatic polymerization [40]. Another study reported mass spectral evidence for the loss of chlorine atoms from 2,4-dichlorophenol during incubation with a laccase from the fungus *Rhizoctonia praticola* [41]. Therefore, it can be concluded that for DCF, the loss of Cl occurred during the oxidative coupling as the chlorines on the ring were in the *ortho*-positions relative to the amino functional group, which made them prone to such a dehalogenation event. This observation is also consistent with the speculation above that oligomerization ensues by C-C coupling. Given the high degree of similarity between DCF and ACF, it is expected that reaction mixtures of the latter would show an analogous coupling pattern and dechlorination.

There was no evidence of dimer and trimer formation. It can be assumed that the absence of these oligomers is because they are more readily converted to the higher oligomers [42], i.e., tetramer, than the conversion of the monomer to more dimers during the enzymatic reaction. There is a precedent for such competing pathways in the HRP-catalyzed oligomerization of phenol—one of the expected dimers, 4-phenoxyphenol, was not found in the reaction mixture because its pseudo-first-order rate constant for conversion by the enzyme was 160-fold higher than that of the parent phenol [42]. The higher oligomers arising here with secondary, bisaryl anilines is to be contrasted with the dead-end azo-dimers found with primary anilines in other work [35,36,37].

## 3. Materials and Methods

### 3.1. Chemicals and Equipment

DCF, ACF, bovine liver catalase, sodium phosphate, monobasic and dibasic, sodium acetate, potassium chloride, hydrochloric acid, anhydrous ethanol, isopropanol, phenol, HPLC grade methanol, acetonitrile (ACN) and water, were purchased from Sigma Aldrich Chemical Company Inc. (Oakville, ON, Canada). Crude solid SBP was obtained from Organic Technologies (Coshocton, OH, USA). Liquid *Arthromyces ramosus* peroxidase (ARP) concentrate was obtained from Novozymes (Franklinton, NC, USA). 4-Aminoantipyrine (AAP) was procured from BDH Inc. (Toronto, ON, Canada). H_2_O_2_ (30% pure) was purchased from ACP Chemicals Inc. (Montreal, QC, Canada). Syringe filters (0.2 μm, nonsterile) were obtained from Sarstedt (Montreal, QC, Canada) and 10 mL syringes were purchased from Fischer Scientific Company (Ottawa, ON, Canada).

Solution pH was measured with an Oakton PC 700 pH meter obtained from Cole-Parmer (Quebec, QC, Canada). The pH meter was calibrated before each use with at least two standard buffer solutions (pH 4.00, 7.00, and 10.00) to establish a reliable calibration curve. The electrode was rinsed thoroughly between measurements, and periodic performance checks were conducted to ensure consistent readings. If drift or inaccuracies were observed, recalibration was performed. A 1000 μL quartz glass spectrophotometer cuvette, with a 1 cm path length (104-QS type) from Hellma Analytics (Concord, ON, Canada) was used for spectroscopic measurements. Pipettors used in sample preparation were regularly calibrated through gravimetric analysis with a precision balance. Pipettes were tested at multiple volume settings, and deviations were compared to manufacturer specifications. Any deviations beyond the acceptable tolerance were corrected through recalibration or replacement. An Agilent (Mississauga, ON, Canada) UV-Vis spectrophotometer, model 8453, with λ range of 190–1100 nm and 1 nm resolution controlled by a Hewlett Packard Vectra ES/12 computer was used to determine solution absorbances (λ_max_) of the substrates and to test SBP enzyme activity. Routine performance checks and wavelength accuracy tests were conducted periodically to ensure reliable absorbance measurements. Baseline corrections and instrument validation procedures were followed as per manufacturer recommendations. The residual substrate concentrations after enzymatic treatment were quantified using a high-performance liquid chromatography (HPLC) system (Waters Corp., Mississauga, ON, Canada) with a model 2489 dual wavelength absorbance detector, model 1525 binary HPLC pump, and model 2707 autosampler. A C18 (5 µm, 4.6 × 150 mm) Symmetry column (also from Waters), was used for this study. The HPLC was operated using Breeze 2.0 software. The HPLC system was calibrated and validated using a multi-step approach, including the generation of a standard calibration curve with known concentrations of the target analytes, ensuring a linear response across the expected concentration range. System suitability tests, including resolution, peak symmetry, and repeatability, were conducted before each analytical run. Periodic injections of quality control samples were performed to verify system stability and reproducibility. HPLC conditions were for diclofenac, 30% formic acid (0.1%)/70% ACN, flow rate 1.0 mL/min, λmax 276 nm; and for aceclofenac, 30% formic acid (0.1%)/70% ACN, flow rate 1.0 mL/min, λmax 275 nm.

MS analysis was carried out at Queen’s University, Kingston, ON using a Thermo Scientific (Mississauga, ON, Canada) Orbitrap Velos Pro (Easy-nLC/HESI Hybrid Ion Trap-Orbitrap Mass Spectrometer) or an Agilent (Mississauga, ON, Canada) AdvanceBio 6545XT LC/QTOF (1260 Infinity II LC APCI/ESI Quadrupole Time of Flight Mass Spectrometer) in high-resolution mode. Data acquisition was performed either in the positive- or negative-ion mode. The acquired mass spectra were subjected to qualitative analysis for molecular formulae targeting possibly formed oligomers and oligomer derivatives with a 10 ppm difference (between measured and calculated masses) used as the cutoff for unambiguously linking a given mass to a specific chemical formula. In electrospray ionization (ESI), the analyte is pumped to a capillary and a high voltage is applied, which makes the droplets spray from the tip of the capillary and evaporate. The evaporation process is also supported by heat and a nebulizing gas, nitrogen. The gas-phase ions then enter the mass spectrometer for detection as their mass-to-charge ratio, *m*/*z*. The acquisition range of the probe was 50 to 2000 mass-to-charge ratio (*m*/*z*).

### 3.2. Enzyme Stock Solutions and Buffers

The SBP stock solution was prepared by mixing 1.4 g of crude solid enzyme with 100 mL distilled water at low speed (approximately 400 rpm) for 24 h. The suspension was centrifugated at 4000 rpm for 25 min and the supernatant was taken as the stock solution and stored at 4 °C.

Solid bovine liver catalase (0.5 g) was dissolved in 100 mL of distilled water (995 U/mL). The solution was magnetically stirred for approximately 3 h before being stored at 4 °C for later use. Citrate–phosphate buffers were freshly prepared to be used in the range of pH 3.0–8.0 [43].

### 3.3. SBP Activity Assay

A colorimetric assay [16] was used to measure the SBP activity. Enzyme activity is defined in standard catalytic units (U), where 1.0 unit of SBP is the amount of enzyme needed to convert 1.0 µmol of H_2_O_2_/min under the assay conditions. Enzyme activity was measured by monitoring the initial rate of color formation of a pink chromophore at 510 nm, generated by oxidative coupling of phenol (10 mM) and 4-aminoantipyrine (4-AAP; 2.4 mM) in the presence of H_2_O_2_ (0.20 mM) with SBP as catalyst.

### 3.4. Experimental Protocols

The enzymatic reactions were conducted in 30 mL open batch reactors, in triplicate and at room temperature (22 ± 1 °C). The 20 mL reaction medium consisted of 40 mM buffer, 0.1 mM of target compound, SBP, and hydrogen peroxide in appropriate concentrations. Hydrogen peroxide was added last to the reaction mixture to start the reaction. After 3 h of mixing, the reaction was quenched immediately by adding 100 μL catalase stock solution to consume residual hydrogen peroxide. The sample was then microfiltered with a pre-conditioned, 0.22 µm PES syringe filter, and the residual substrate concentration was analyzed by HPLC. For each substrate, three control experiments were conducted, one without SBP, one without H_2_O_2_, and the last without SBP and H_2_O_2_.

pH, SBP activity, H_2_O_2_ concentration, and reaction time were the parameters studied for enzymatic treatment. The error bars demonstrate the standard deviations of triplicate measurements; error bars are not visible for the data points with very small deviations. For the effect of pH, the reactions were conducted at different pHs under stringent conditions (limited SBP, so that the effect of pH can be clearly discerned). Subsequently, at the most effective pH, the reactions were formulated for sensitivity to [H_2_O_2_] and SBP activity to reach 95% removal. Reaction times of experiments were also measured by taking 5 mL samples from a 75 mL batch reactor at selected time intervals, quenching them with catalase, filtering them, and analyzing the filtrates by HPLC. For preliminary identification of plausible polymerization products by mass spectrometry, reactions were carried out under the most effective conditions and unfiltered samples were submitted.

## 4. Conclusions

Crude seedcoat SBP was proficient in catalyzing the conversion of DCF and ACF, as hypothetically emerging contaminants, in an ecologically friendly and sustainable manner. With SBP’s availability in massive quantity from a by-product of commodity processing of the world’s largest seed crop, SBP-based treatment of selected compounds in this study provides a cost-effective alternative to conventional treatment processes for removing them from wastewater at >95% removal efficiency. The SBP and H_2_O_2_ requirements for such removal of these compounds were much lower than for many other aromatic compounds studied previously. The only products detected in solution were various forms of the oxidative tetramer, which provides valuable insights into the mechanism of product formation. Future work will seek to extend the treatment to realistic concentrations of DCF and ACF in sewage treatment effluents.

## Figures and Tables

**Table 1:** Half-lives and normalized half-lives of various SBP substrates *.

	Reference	Substrate	Half-Life (min)	Normalized Half-Life (min.U/mL)
Present Study	-	DCF	1.43 ± 0.01	0.22 ± 0.02
		ACF	0.84 ± 0.05	0.49 ± 0.01
Sulfa Drugs	[35]	Sulfamethoxazole	0.804 ± 0.003	0.0804 ± 0.0003
		Sulfamerazine	1.22 ± 0.01	0.061 ± 0.008
HACs ^†^	[36]	Pyrrole	49.2 ± 3.0	246 ± 15
		Indole	25.2 ± 1.2	11.3 ± 0.5
		2-Aminothiazole	33.0 ± 0.6	132 ± 2
		2-Aminobenzothiazole	720 ± 0.01	3240 ± 0.0
		4-Aminoantipyrine	60.6 ± 1.2	6.06 ± 0.12
		Hydroxybenzotriazole	41.4 ± 1.8	4.97 ± 0.22
		2-Aminoimidazole	5.1 ± 0.2	7.7 ± 0.3
		2-Amino-benzimidazole	29.4 ± 0.6	88.2 ± 1.8
		3-Aminopyrazole	36.0 ± 1.2	108 ± 4
	[32]	3-Hydroxyquinoline	11.9 ± 0.6	1.19 ± 0.06
		3-Aminoquinoline	15.0 ± 0.6	67.5 ± 2.7
Arylamines	[37]	4,4′-Methylenebis (2-chlororaniline)	4.08 ± 0.02	0.408 ± 0.002
		4-Chloro-*o*-toluidine	11.5 ± 0.0	0.104 ± 0.0
	[26]	p-Cresidine	1.80 ± 0.02	0.072 ± 0.001
		4,4′-Oxydianiline	12.4 ± 0.0	0.124 ± 0.0
	[29]	4,4′-Thiodianiline	0.513 ± 0.007	0.0770 ± 0.0011
		4,4′-Methylene-dianiline	0.58 ± 0.10	0.40 ± 0.07
Pesticides	[33]	Bromoxynil	3.00 ± 0.13	2.7 ± 0.02
		Ioxynil	0.51 ± 0.018	0.18 ± 0.01
Azo dyes	[38]	CI Acid Blue 113	8.8 ± 0.60	13.2 ± 0.9
		CI Direct Black 38	2.1 ± 0.2	6.4 ± 0.6
Heteroaromatics	[34]	3-Hydroxycoumarin	12.4 ± 0.5	0.0257 ± 0.0010
		2-Aminobenzoxazole	129 ± 4 (0.44)	452 ± 15
Dyes	[39]	*p*-Anisidine	5.46 ± 0.84	0.0097 ± 0.0011
		CI Methyl Orange	7 ± 2	0.048 ± 0.010

* Aside from the top four entries and pesticides, this table is taken from Ziayee Bideh (2021) [35]. ^†^ HACs refers to heterocyclic aromatic compounds.

**Table 2:** Summary of MS results for standard and identified products of SBP-catalyzed reaction of DCF.

Standard	Symbol	Molecular Formula	*m*/*z*	Detected
DCF	MH	C_14_H_11_Cl_2_NNaO_2_	318.00646	*
	M	C_14_H_10_Cl_2_NNaO_2_	316.99863	
Identified products	MH-Na+H	C_14_H_12_Cl_2_NO_2_	296.02451	*
	M_2_H-2	C_28_H_18_Cl_4_N_2_Na_2_O_4_	631.98161	
	M_3_H-4	C_42_H_26_Cl_6_N_3_Na_3_O_6_	946.96459	
	M_4_H-6	C_56_H_34_Cl_8_N_4_Na_4_O_8_	1261.94757	*
	M_4_H-6-4Na+H	C_56_H_35_Cl_8_N_4_O_8_	1170.99631	*
	M_4_H-6-3Cl	C_56_H_34_Cl_5_N_4_O_8_	1065.08193	*

* with all formulae detected, the requisite ^13^C peaks were found and occasionally the ^37^Cl were found.

## Data Availability

All data supporting the reported results are in the manuscript.
